# Supplementary material for: A novel RNA pol II CTD interaction site on the mRNA capping enzyme is essential for its allosteric activation
Source: Nucleic Acids Res. 2021 Mar 3;49(6):3109–26. doi: 10.1093/nar/gkab130 (PMC8034621; doi:10.1093/nar/gkab130)
Supplement: gkab130_Supplemental_Files [file gkab130_supplemental_files.zip › Supplementary_R1.pdf]

A Novel RNA Pol II CTD interaction site on the mRNA Capping Enzyme is essential for its allosteric activation

Marcus G. Bage[1,2]*, Rajaei Almohammed[2], Victoria H. Cowling[2], Andrei V. Pisliakov[1,3]*

[1]Computational Biology, School of Life Sciences

[2]Centre for Gene Regulation and Expression, School of Life Sciences

[3]Physics, School of Science and Engineering,

University of Dundee, Dundee, DD1 5EH, UK

*Corresponding authors

Tel: +44 1382 388758; Email: a.pisliakov@dundee.ac.uk

Correspondence may also be addressed to Marcus G. Bage.
Email: m.bage@dundee.ac.uk

Supplementary Data:

- 1 Supplementary Table
- 14 Supplementary Figures
- 3 Supplementary Movies

Table S1. Summary of the molecular dynamics simulations performed in this work

	CE GTase	CTD	cMD	aMD
1	human	-	3x 200 ns	3x 1000 ns
2	PBCV-1	-	3x 200 ns	-
3	human	1 heptad, pSer5 and pSer2	3x 200 ns	3x 200 ns
4	human	4 heptads, unphos, extended from N-ter	3x 200 ns	3x 200 ns
5	human	4 heptads, unphos, extended from C-ter	3x 200 ns	3x 200 ns
6	human	4 heptads, pSer5, extended from N-ter	3x 200 ns	3x 200 ns
7	human	4 heptads, pSer5, extended from C-ter	3x 200 ns	3x 200 ns
8	human	4 heptads, pSer2, extended from N-ter	3x 200 ns	3x 200 ns
9	human	4 heptads, pSer2, extended from C-ter	3x 200 ns	3x 200 ns
10	human	~2.5 heptads, pSer5, in *C. albicans* conform.	3x 200 ns	3x 200 ns
11	human	4 heptads, pSer5, from *C. albicans* conform.	3x 50 ns	-
12	human	System 6 final conform., dephos.	3x 50 ns	-
13	human ΔCDS1	System 6 final conform., pSer5	3x 50 ns	-
14	human ΔCDS2	System 6 final conform., pSer5	3x 50 ns	-
15	human ΔCDS1/CDS2	System 6 final conform., pSer5	3x 50 ns	-
16	human	4 heptads, pSer5, extended from N-ter, CDS1+2 bound	3x 200 ns	3x 1000 ns
17	human K294A	-	3x 200 ns	3x 1000 ns
		Total:	7.95 μs	13.80 μs

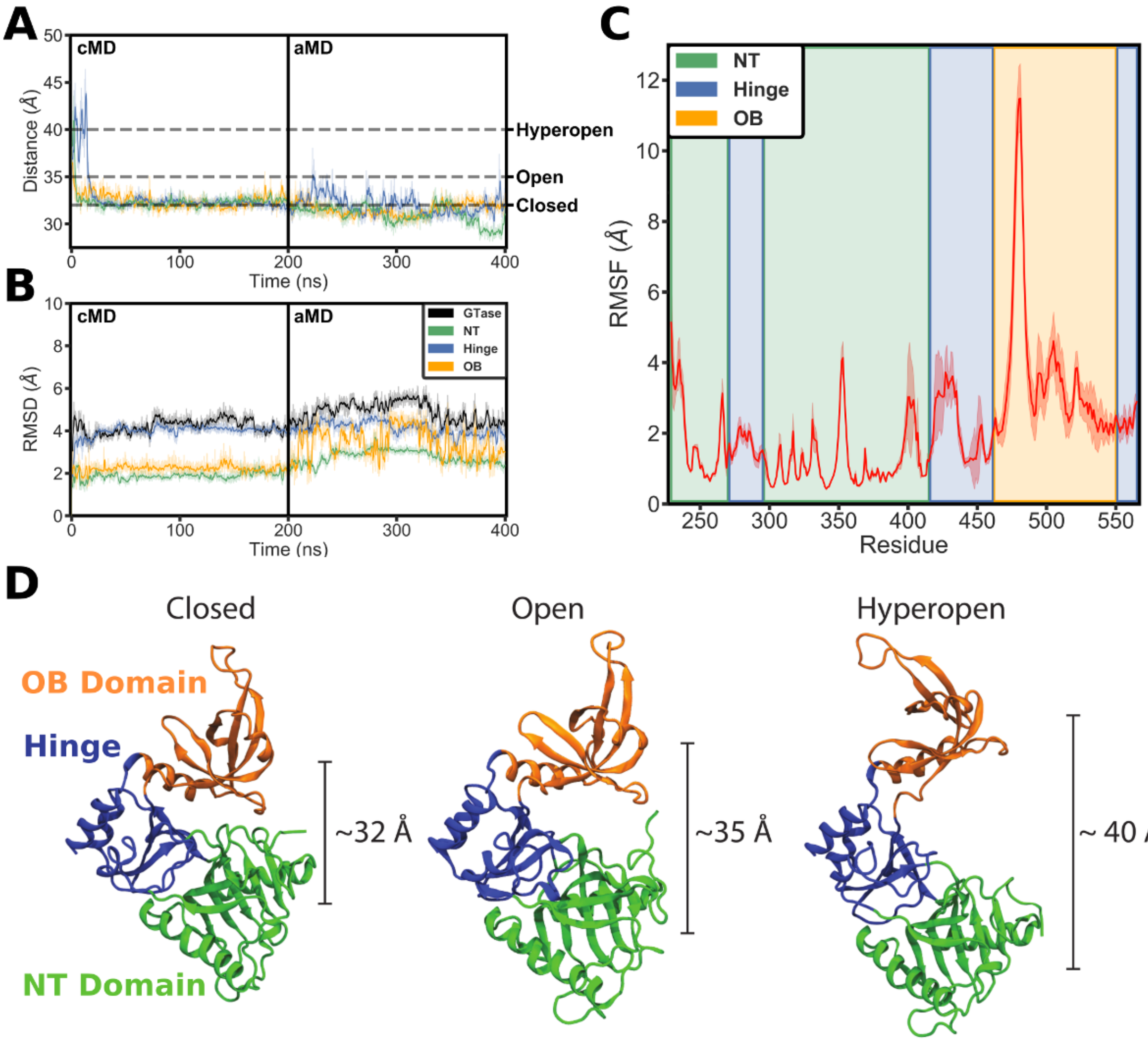

Figure S1

Global conformational dynamics of the *apo* human CE GTase. (**A**) The inter-domain distance (centre-of-mass separation) between the OB domain and NT domain over the duration of the cMD and aMD simulations. Three replicates are shown in orange (replicate 1), blue (replicate 2) and green (replicate 3). (**B**) Backbone RMSDs of the whole GTase domain and its sub-domains during the cMD and aMD trajectories. The results are shown for replicate 1; similar results were obtained for two other replicates. The first frame of the cMD was used as a reference. (**C**) C_α atom RMSFs of the whole GTase domain obtained from aMD simulations. RMSF values represent the mean of the three aMD replicates. The NT domain in the first frame of the cMD was used as a refer-

ence. The shaded area represents one standard deviation. Sub-domains of the GTase structure are highlighted by coloured regions. (**D**) The conformational states that can be adopted by the *apo* CE GTase are designated as ‘Closed’ (separation between the centres of mass of the OB and NT domains ~32 Å), ‘Open’ (~35 Å) and ‘Hyperopen’ ($>$39 Å), in line with the definitions for the PBCV1 GTase by Swift *et al.* [1].

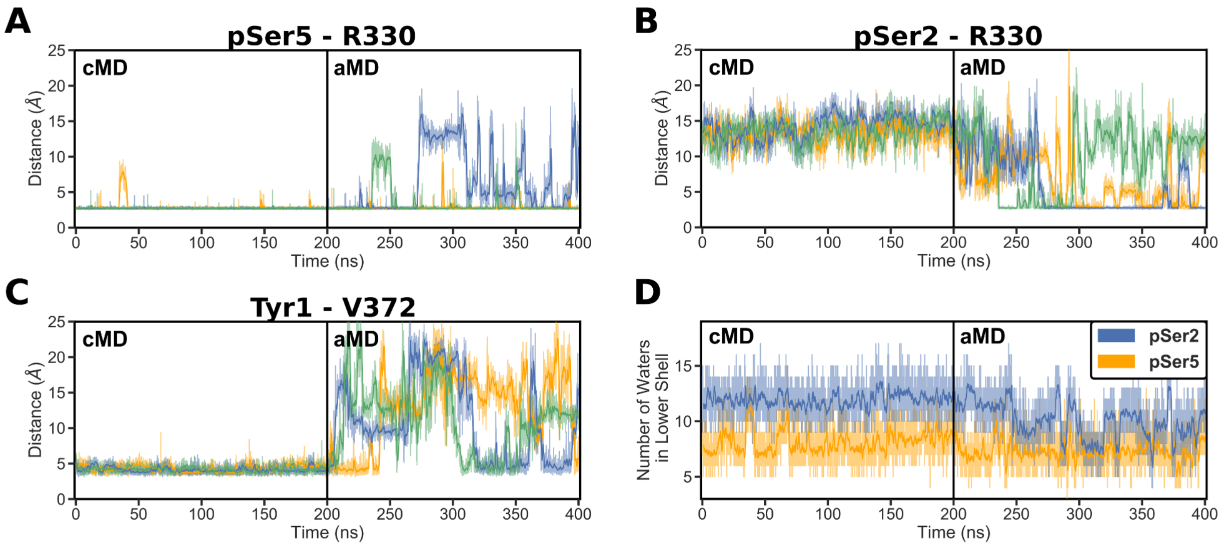

Figure S2

Characterisation of the GTase-CTD interaction sites in the 1-heptad CTD simulation (Ser2 and Ser5 phosphorylated; System 3). (**A-B**) Time-evolution of the minimum distances between, CDS1 (taken as the R330 sidechain nitrogens) and pSer5 (A) or pSer2 (B) of the CTD (taken as the phosphate oxygens). (**C**) Time-evolution of the minimum distance between CDS-Y1 (V372 Cγ atoms) and Tyr1 (sidechain ring) of the CTD. Replicates in (A-C) are displayed in orange (replicate 1), blue (replicate 2) and green (replicate 3). (**D**) Solvent exposure of the pSer2 and pSer5 groups in replicate 1. Number of waters in the lower solvation shell ($<$ 3.4 Å) of the phosphate group is plotted over the cMD and aMD simulation trajectories.

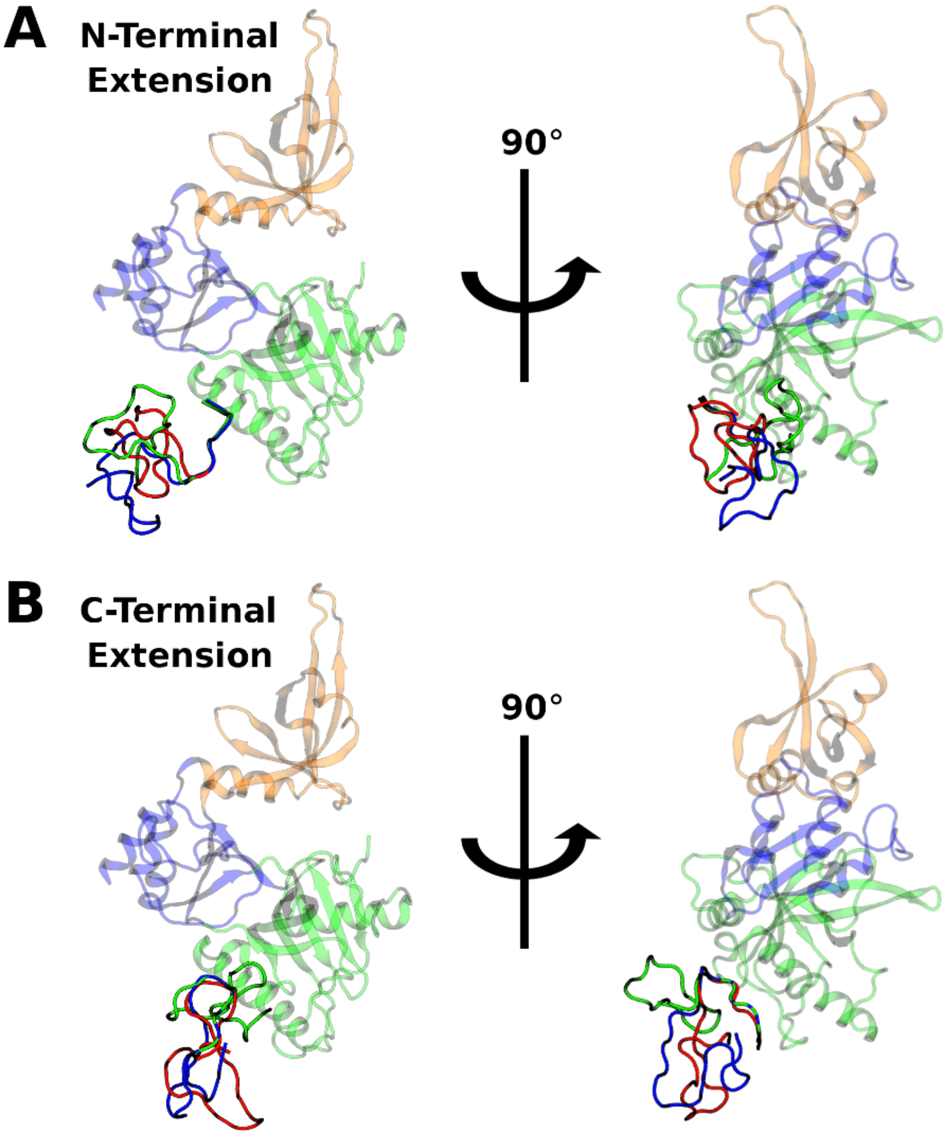

Figure S3

Starting CTD conformations in the simulations with a 4-heptad CTD. Each of the three replicates used a different starting structure of the CTD peptide, which were generated using the PEP-FOLD server [2], extending the CTD either in the N-ter direction (**A**) or in the C-ter direction (**B**) (see details in the Methods). The CTD conformations in the three replicates are shown in red (replicate 1), blue (replicate 2) and green (replicate 3).

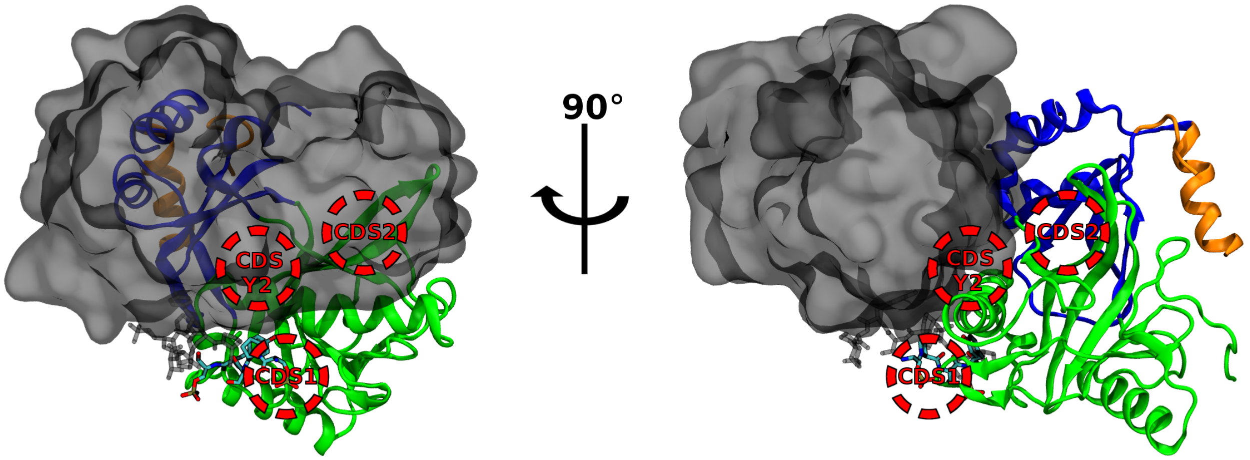

Figure S4

Obstruction of CDS2 and CDS-Y2 sites in the previously reported cocrystal structure of the mouse CE GTase-CTD complex. Structure described by Ghosh *et al.* (PDB ID: 3RTX) [3]. CTD access to the CDS2 and CDS-Y2 sites is blocked by the artificial homodimer interface in the asymmetric unit. The first monomer of the GTase is displayed in the cartoon representation, the second monomer is displayed with a transparent grey surface, and the CTD is shown in stick representation. CDS1, CDS2 and CDS-Y2 locations are indicated in red.

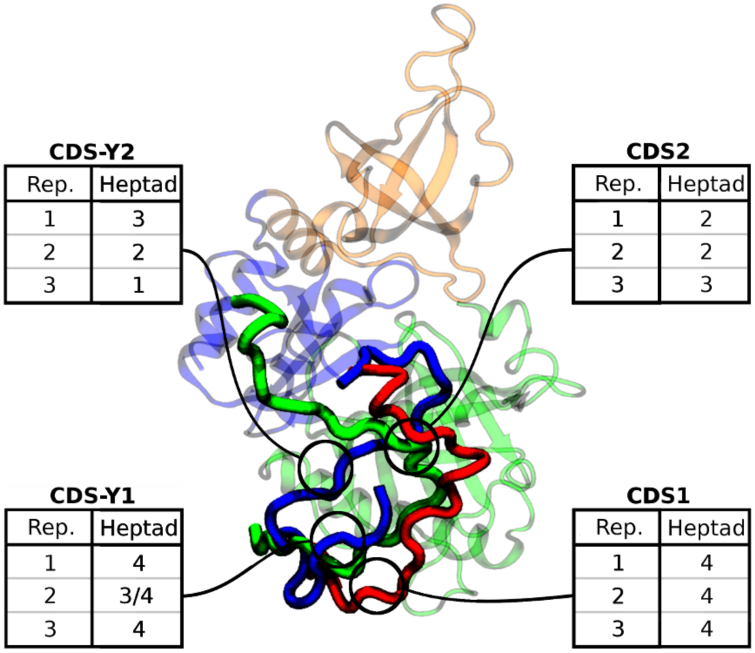

Figure S5

CTD peptide binding conformations and looping out mechanism. The CTD conformations are taken from the final aMD snapshots of the 4-heptad (N-ter extended), pSer5 CTD system (System 6), with the CTD in replicate 1 displayed in red, replicate 2 in blue, and replicate 3 in green. The four CDS sites are labelled by circles. The boxes indicate which heptad predominantly interacts with the respective site in each simulation replicate. CTD heptads are numbered from the N-terminus (1) to the C-terminus (4).

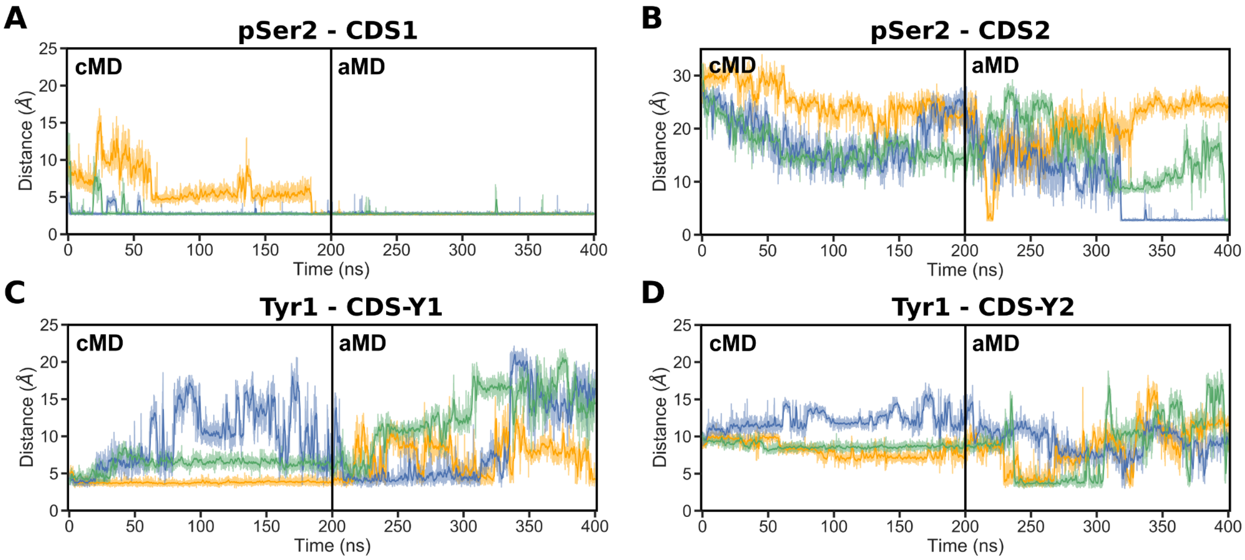

Figure S6

Time-evolution minimum distances showing the occupation of each CDS site by the respective pSer2 CTD group over the duration of the cMD and aMD simulations of System 9 (4-heptad, pSer2 CTD extended in the C-ter direction). Distances obtained in three replicates shown as orange (replicate 1), blue (replicate 2) and green (replicate 3). The occupation of each site was described by taking representative sidechain minimum distances as follows: (**A**) CDS1, R330 sidechain nitrogens to the pSer2 phosphate oxygens, (**B**) CDS2, R411 sidechain nitrogens to the pSer2 phosphate oxygens, (**C**) CDS-Y1, V372 Cγ atoms to the Tyr1 ring, and (**D**) CDS-Y2, L381 sidechain to the Tyr1 ring.

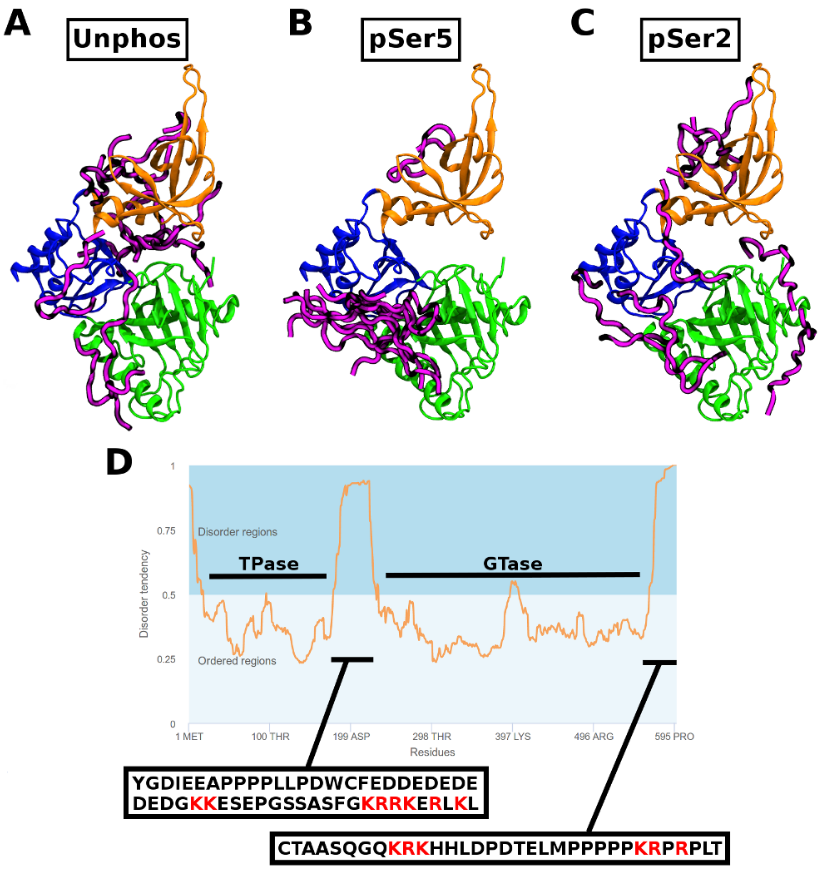

Figure S7

Exploring potential CTD interaction sites in regions distal from the NT domain. (**A-C**) Top 10 ranking models predicted by the PIPER-FlexPepDock server showing the binding conformations of the 2-heptad CTD (magenta) in the unphosphorylated (A), Ser5 phosphorylated (B), and Ser2 phosphorylated (C) states. Glutamic acid was used as a phosphomimetic. In the unphosphorylated state, the CTD peptide forms interactions with many regions of the GTase (likely unspecific). The pSer5 phosphorylated peptide docks specifically to the NT domain in the region sampled during our MD simulations, indicating the preferred localisation of pSer5 CTD. The PIPER-FlexPepDock method also identified pSer5 interactions with the novel CDS2 interaction site in 4 of the top 10 models. The pSer2 peptide was seen docked to the CDS2 site in 2 of the top 10 models, but in general can dock into a much wider number of regions on the GTase compared to the pSer5 CTD. (**D**) Disorder prediction for the whole human Capping Enzyme sequence as obtained from the MetadisorderMD2 server [4]. Regions predicted to be disordered can be seen at both the N- and

C-terminal sides of the GTase domain (these were not resolved in any of the crystal structures). The amino acid sequences of these regions are displayed in boxes, and positively charged residues are highlighted in red.

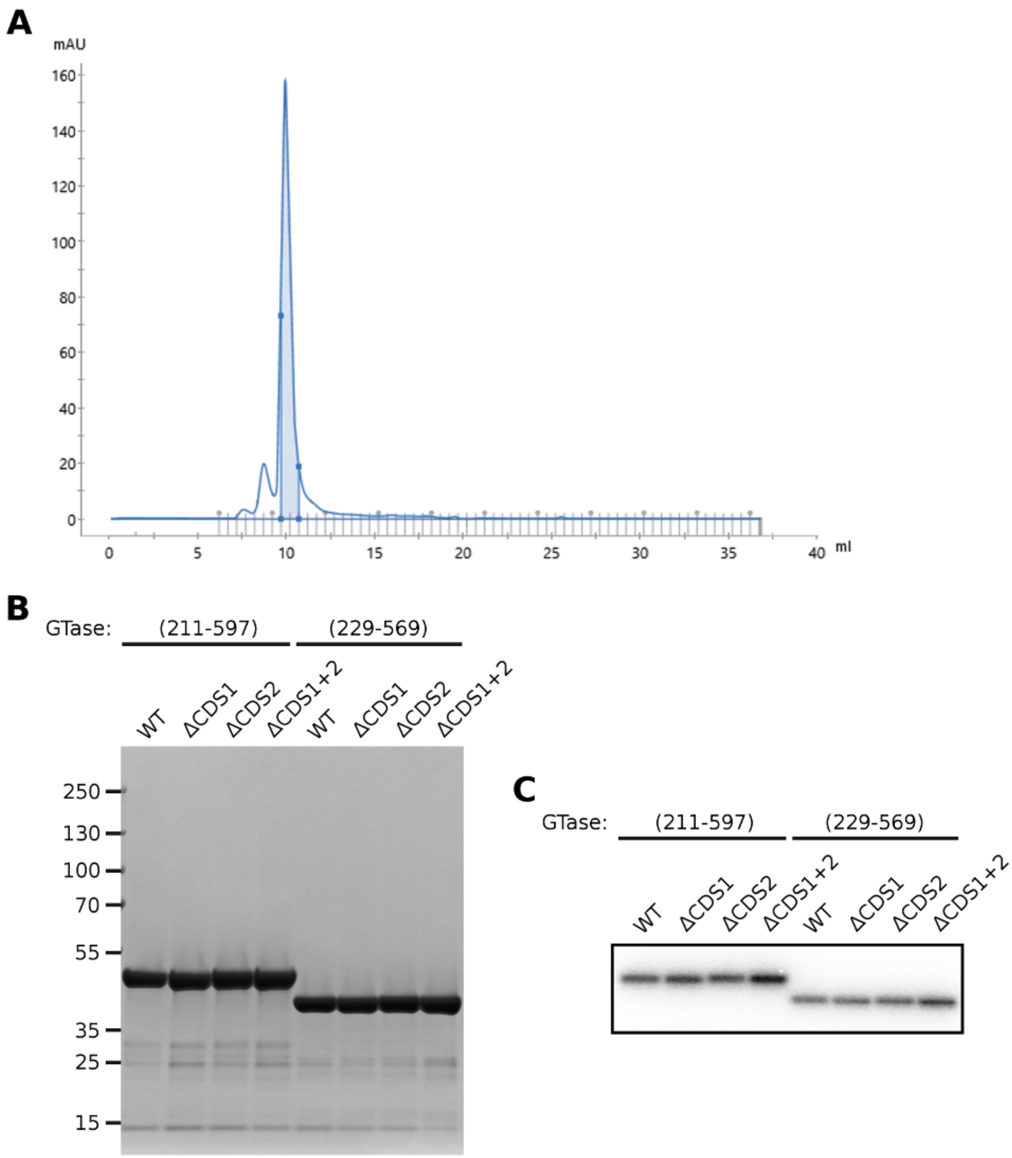

Figure S8

Purity and basal activity of the human CE GTase constructs. (**A**) UV chromatograph of the size-exclusion chromatography stage of purification showing a representative elution profile (WT human CE GTase, residues 211-597), with the pooled fractions highlighted in blue. (**B**) Coomassie-stained SDS-PAGE gel of all constructs after the purification process. (**C**) Basal guanylyltransferase activity assay of all constructs.

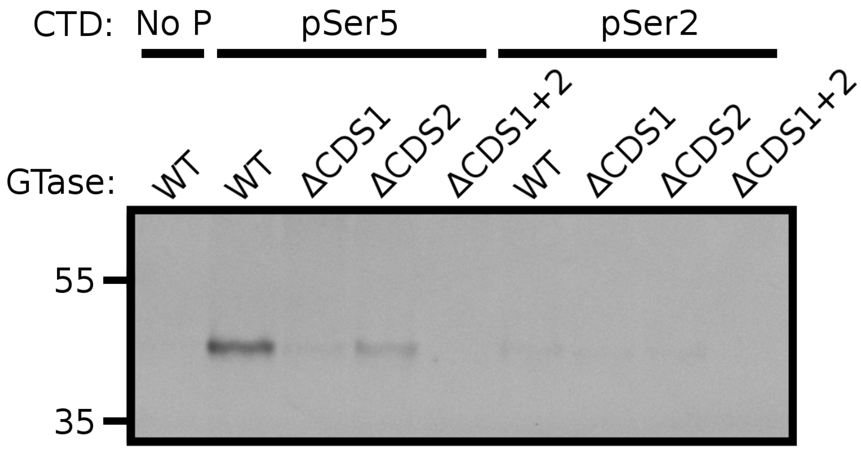

Figure S9

Pull-down assay testing the CTD binding affinity in the wild-type and mutant core GTase (229-569) for the unphosphorylated, pSer5 and pSer2 CTD peptides. Recombinant human CE GTase was incubated with biotinylated peptides of 4 CTD heptads, in either their unphosphorylated (no P CTD) or phosphorylated state (pSer5 or pSer2 CTD) bound to streptavidin-coupled Dynabeads. The level of GTase binding to the CTD peptides was assessed by SDS-PAGE stained with Coomassie Blue.

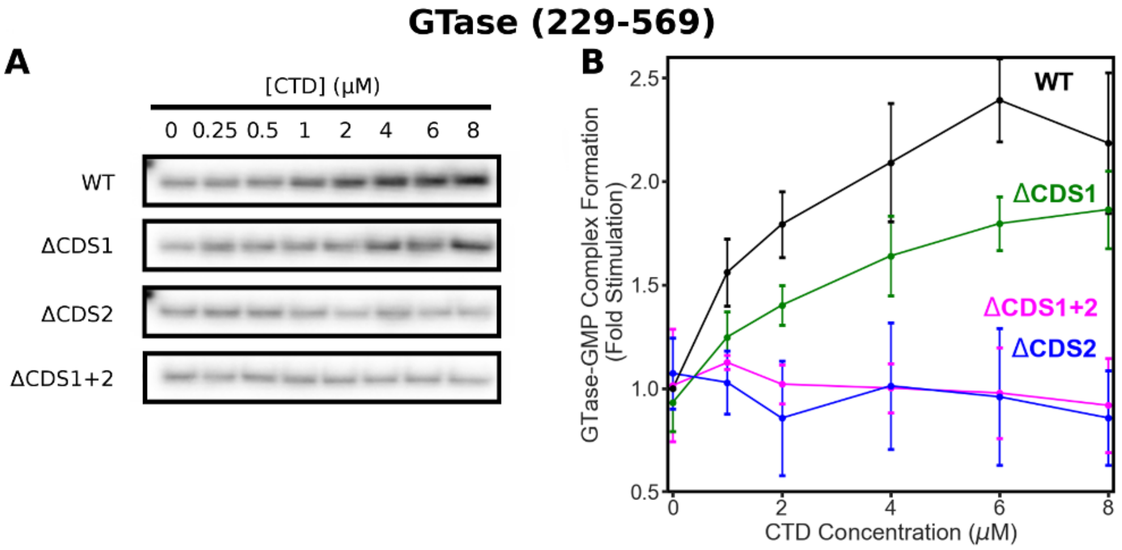

Figure S10

GTase activity assays characterising the CTD activation effect for the core GTase (229–569) mutants. Band quantification was performed and normalised relative to the basal WT GTase, with error bars denoting one standard deviation. Compare with the results for the 211-597 GTase variant containing the flanking regions (Figures 6F and 6G in the main text).

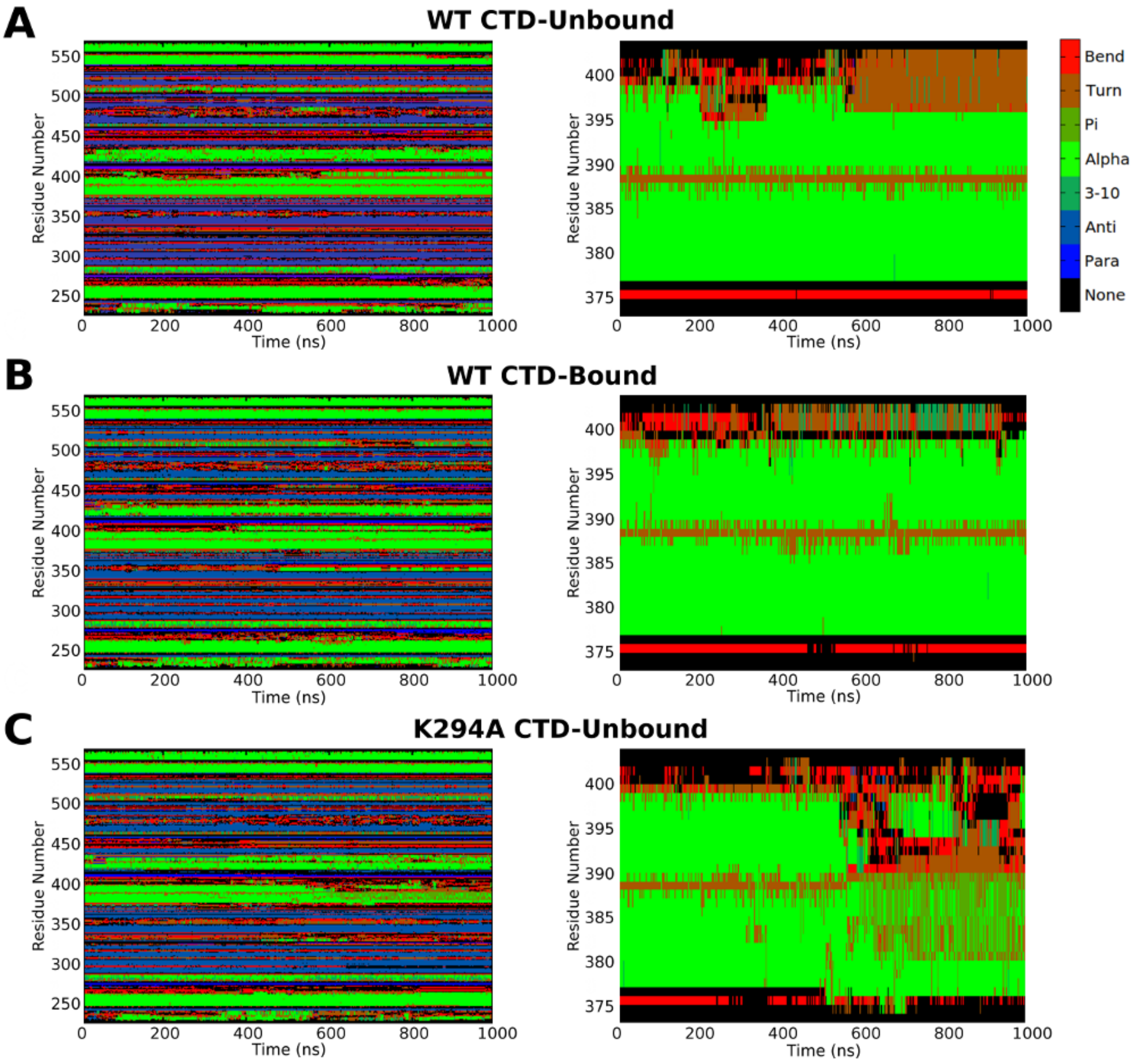

Figure S11

Comparison of the secondary structures between the CTD-unbound, CTD-bound, and the K294A aMD simulations (Systems 1, 16 and 17). (**A-C**) DSSP analysis of the WT GTase no-CTD system (A; System 1), the WT GTase 4-heptad pSer5 N-ter extended CTD-bound system (B; System 16), and the K294A CTD-unbound system (C; System 17) over one 1 μs aMD replicate. The whole GTase DSSP analysis (left) and the helix αC region (residues 373-403) analysis (right) are displayed.

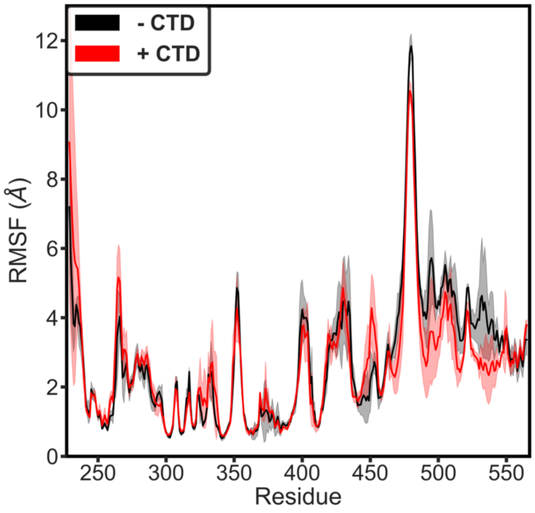

Figure S12

Conformational dynamics of the GTase in the presence and absence of the CTD. C_{α} atom RMSFs were calculated for the no-CTD (System 1, **black**) and 4-heptad pSer5 CTD-bound (N-ter extended; System 16; **red**) GTase systems. RMSF values represent the mean of the three 1 μs long aMD replicates. The NT domain in the first frame of the cMD was used as a reference. The shaded area around the curve represents one standard deviation.

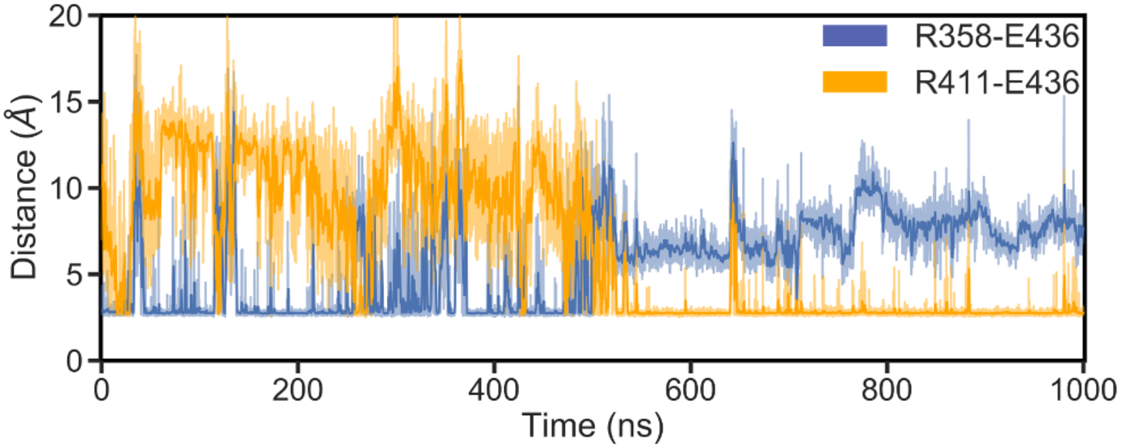

Figure S13

Exchange between the salt bridges R358-E436 (blue) and R411-E436 (yellow) in the K294A GTase aMD simulation (the same replicate that is displayed in Figures 7B, C and E). The distances are obtained as the minimum distance between the corresponding sidechains.

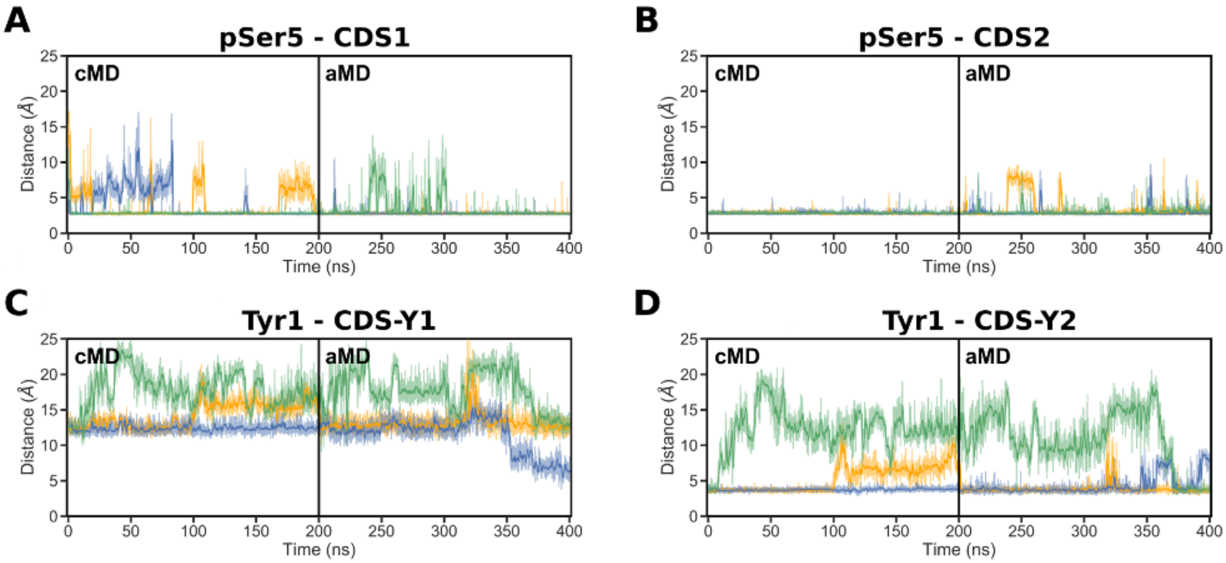

Figure S14

The GTase-CTD interaction in simulations started with the pSer5 CTD in the *C. albicans* starting conformation (the 'yeast' orientation, System 10). (**A-D**) Time-evolution of the minimum distances showing the occupation of each CDS by the respective pSer5 CTD group over the duration of the cMD and aMD simulations. The distances are defined as in Figure 3 in the main text. Distances obtained in three replicates are shown in orange (replicate 1), blue (replicate 2) and green (replicate 3). The distance between the CDS1 and pSer5 phosphate in the *C. albicans* GTase-CTD cocrystal structure is only ~11.5 Å. One pSer5 group of the CTD was seen to occupy the 'mammalian' CDS1 site within 20 ns of the simulations and this interaction remained stable. This suggests that the locations of the 'mammalian' and 'yeast' CDS1 sites are close enough on the GTase surface to perform the same underlying role of CTD binding. The CDS2 site remains occupied for the duration of the simulations.

Movies

Movie S1 — Overview of the CTD interaction sites observed during the simulations of the GTase with the 4-heptad pSer5 CTD extended in the N-ter direction (System 6, replicate 1).

Movie S2 — pSer5 binding and the residues involved in the CDS2 interaction site.

Movie S3 — Residues involved in the CDS-Y2 interaction site.

1 References

1. Swift, R. V. and McCammon, J. A. (2009) Substrate induced population shifts and stochastic gating in the PBCV-1 mRNA capping enzyme. *Journal of the American Chemical Society* **131**, 5126–5133.

2. Lamiable, A., Thévenet, P., Rey, J., Vavrusa, M., Derreumaux, P. and Tufféry, P. (2016) PEP-FOLD3: faster de novo structure prediction for linear peptides in solution and in complex. *Nucleic Acids Research* **44**, W449–W454.

3. Ghosh, A., Shuman, S. and Lima, C. D. (2011) Structural insights to how mammalian capping enzyme reads the CTD code. *Molecular Cell* **43**, 299–310.

4. Kozlowski, L. P. and Bujnicki, J. M. (2012) MetaDisorder: a meta-server for the prediction of intrinsic disorder in proteins. *BMC Bioinformatics* **13**, 111.
